# Nutrition in calcium nephrolithiasis

**DOI:** 10.1186/1479-5876-11-109

**Published:** 2013-05-01

**Authors:** Elena Dogliotti, Giuseppe Vezzoli, Antonio Nouvenne, Tiziana Meschi, Annalisa Terranegra, Alessandra Mingione, Caterina Brasacchio, Benedetta Raspini, Daniele Cusi, Laura Soldati

**Affiliations:** 1 Fondazione Umberto Veronesi, p.zza Velasca 5, 20142, Milano, Italy; 2 Department of Health Sciences, Università degli Studi di Milano, via A. Di Rudini 8, 20142, Milano, Italy; 3 Nephrology Unit, San Raffaele Hospital, via Olgettina 60, Milano, Italy; 4 Department of Clinical and Experimental Medicine, University of Parma, via A. Gramsci 14, 43126, Parma, Italy

**Keywords:** Nephrolithiasis, Nutrition, Calcium, Stones prevention

## Abstract

Idiopathic calcium nephrolithiasis is a multifactorial disease with a complex pathogenesis due to genetic and environmental factors. The importance of social and health effects of nephrolithiasis is further highlighted by the strong tendency to relapse of the disease. Long-term prospective studies show a peak of disease recurrence within 2–3 years since onset, 40-50% of patients have a recurrence after 5 years and more than 50-60% after 10 years. International nutritional studies demonstrated that nutritional habits are relevant in therapy and prevention approaches of nephrolithiasis. Water, right intake of calcium, low intake of sodium, high levels of urinary citrate are certainly important for the primary and secondary prevention of nephrolithiasis.

In this review is discussed how the correction of nutritional mistakes can reduce the incidence of recurrent nephrolithiasis.

## Background

Idiopathic nephrolithiasis is a disease that has been widely spread in countries with high socio-economic levels along with other conditions such as obesity, hypertension, atherosclerosis, cardiovascular disease and type 2 diabetes [1].In these countries the prevalence of nephrolithiasis is about 6-9% in men and 3-4% in women [2,3]. Among the various types of stones, idiopathic calculi of calcium-oxalate and/or calcium-phosphate are the most frequent form with a result of around 80% [4-7]. The importance of social and health effects of nephrolithiasis is further highlighted by the strong tendency to relapse of the disease [2,8-10]. Long-term prospective studies show a peak of disease recurrence within 2–3 years since onset, 40-50% of patients have a recurrence after 5 years and more than 50-60% after 10 years [2]. The most affected are usually males [11,12], but in recent decades the frequency of the disease is increasing, especially among women [13,14] and today the sex ratio approaches unity [15]. The trend of prevalence is also increasing in developing countries such as China, India and Brazil in relation to changes in lifestyle [16]. It is believed that the calcium nephrolithiasis is a multifactorial disease of complex pathogenesis, whose onset is due to multiple genetic and environmental factors [16-18].

The distribution of nephrolithiasis family does not prove a classic Mendelian transmission [19]. It is assumed, therefore, that multiple genes are involved, and their combination can produce in single individuals a specific predisposition to nephrolithiasis. However, genetic predisposition to nephrolithiasis can be modified by environmental factors, among which nutritional factors have an important role. These factors may modulate the lithogenic effects of gene products, but also change the same gene expression through specific mechanisms called “epigenetic”. The discovery that the provision of specific nutrients can alter gene transcription has partly reversed the concepts of classical genetics giving a new and relevant importance to nutrients [20].

The analysis of dietary patterns has pointed out how the increased prevalence of stones in countries such as the United States went in parallel with the increased size of the portions consumed both at home and outside, along with the increased sedentary lifestyle and obesity, which has increased from 14.5% in 1971 to 30.9% in 1999 [21,22]. The same trend is observed in European northern countries, while in the countries of southern Europe there has been a gradual abandonment of the Mediterranean diet in the younger generations as well as their increased sedentary lifestyle and obesity [23].

This review will take into account the nutritional aspects considered most peculiar in calcium nephrolithiasis.

## Nutritional factors

### Water and other fluids

Several studies [14,24,25] have shown that people who consume a quantity of less than 1.5 L/day of fluids undergo kidney stones, accounting for 50% higher than people who assume more than 2.5 L/day of fluids [26]. Two studies, one observational [27] and one of intervention [28], demonstrate the beneficial effects of water intake, both on the prevention of kidney and on recurrences. As a result, it was observed that the relapse rate fell from 27% to 12%, doubling from 1 to 2 L/day the volume of water daily consumed during a follow-up of 5 years.

If water is certainly important for the primary and secondary prevention of nephrolithiasis, it is still controversial whether the hard water with a high content of divalent ions, has the same anti-lithogenic effect of fresh water, with low dry residue [28]. The choice of the water to be consumed should be personalized, considering the overall contribution of trace elements such as calcium, sodium, potassium, chloride, magnesium and trace elements such as iron, fluorine, iodine, zinc, selenium. However, the choice of a oligomineral, low NaCl content water seems to offer a good compromise [29]. As an alternative to water, there are drinks that exert a positive effect since they increase pH and/or citrate and/or volume, such as for example freshly squeezed oranges or lemons, juices made from the same, green tea and wine [30].

Beer has been found to have a protective effect in calcium stone disease while it seems to increase the risk of urate stone disease due to the uricosuric effect caused by its high purine-guanosine content [31,32]. Cranberry juice is not known to protect from calcium nephrolithiasis but is particularly useful in cases of infected stone disease [33,34].

Tea and coffee, on the other hand, increase oxalate even although it has been documented that this effect is cancelled out if milk is added, the calcium of which binds with the oxalate, thereby reducing its absorption [35]. Other drinks are pro-lithogenic, and have a mechanism which has not been fully understood (grapefruit and apple juice, cola) [36]. In this connection, it is important to mention soft drinks, given the quantities and frequency with which they are consumed: recent studies have identified an increased risk of both calcium and uric acid stone disease correlated with the consumption of soft drinks – probably due to their fructose, sucrose and phosphoric acid content – and have documented a reduction in the risk of recurrences of approx. 10% in patients who were instructed to avoid soft drinks [37,38].

### Calcium and sodium chloride

Going from a dietary content of calcium equal to 400 mg/day to one of 1200 mg/day, urinary excretion of calcium increases from 120 mg/day to 180 mg/day in normal subjects and from 240 mg/day to 400 mg/day in hypercalciuric subjects [29,39]. For this reason, the reduction of the consumption of milk and dairy products has been for many years the first measure recommended to stone formers.

With the current knowledge [25,40] this requirement is unfounded for the following reasons: 1) the reduction of calcium in the diet increases the share of oxalate absorbed in the intestine because it prevents the formation of calcium-oxalate complexes which are not absorbable [41]; 2) when we recommend the dietary limitation of milk and dairy products, patients are likely to increase their animal protein intake in the form of meat, fish and poultry, leading to potential unfavorable consequences [29].

Many studies [9,13,14,24] have shown that the risk of nephrolithiasis is lower than about 30% during a diet with an intake of calcium around 1000 mg/day compared to an intake of less than 600 mg/day.

Furthermore, a randomized study conducted for 5 years on male hypercalciuric stone formers has demonstrated that a lower calcium content diet is less effective in the prevention of recurrences than a diet with a normal content of calcium, but with a low intake of salt and proteins [42].

A lot of studies have shown that the high consumption of cooking salt increases the excretion of calcium [10,42-44]. An increase of about 6 g (100 mmol) of NaCl generates an increase of calciuria of 40 mg (1 mmol) in healthy adults and 80 mg (2 mmol) in hypercalciuric subjects forming kidney stones [29,43].

Several studies suggest that the calciuric effect of cooking salt is due to an inhibition of tubular reabsorption of calcium by the sodium and chloride and that this effect is additive to that of acid load promoted by protein*s*[37,45,46], although a recent work has not confirmed these results [47]. The lithogenic risk is further increased by decreased excretion of citrate associated with the acid load and to cellular potassium deficiency [41-44].

### Citrate

The urinary citrate is an inhibitor of the growth and aggregation of the crystals of calcium oxalate and calcium phosphate because it forms stable complexes with free calcium preventing the bond with oxalate and phosphate. This explains why one of the most common metabolic disorders associated with calcium nephrolithiasis is the hypocitraturia. Hypocitraturic patients have been described between 19 and 63% of stone formers in different statistics [48]. The measurement of citrate in the urine has been introduced into the metabolic routine of stone formers and its deficiency is correctable with an oral support of alkali in the majority of patients [48], even if one part of them does not normalize the excretion of citrate with the oral load of alkalis or citrate.

**Figure 1:**
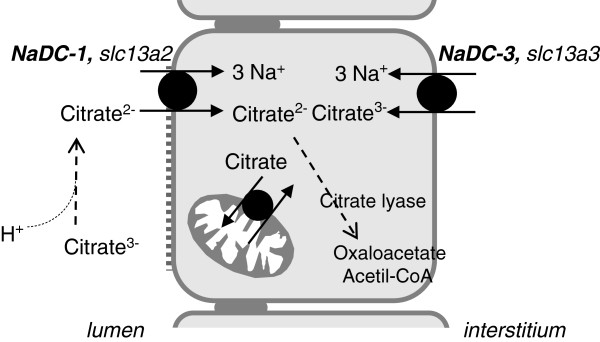
**Schema for the metabolism of citrate in a proximal tubule cell.** The carrier NADC-1 of apical membrane reabsorbs the bivalent citrate. In conditions of acidosis the presence of H^+^ in the proximal tubule fluid stimulates the formation of citrate bivalent from trivalent. The divalent citrate is reabsorbed and metabolized by citrate lyase or through the tricarboxylic acid cycle in the mitochondria of proximal tubular cells. NADC-1 (slc13a2) = Na-dependent low affinity carrier of dicarboxylic acids. NADC-3 (slc13a3) = Na-dependent high affinity carrier of dicarboxylic acids.

The main cause for hypocitraturia is metabolic acidosis, in its various forms. It works by promoting the formation of divalent citrate in the proximal renal tubule. The bivalent form is more easily reabsorbed in the proximal tubule (Figure 1) through a specific transporter (NADC-1) that is also expressed in the intestine (small intestine and colon). Moreover a NADC-1 defect has been associated to nephrolithiasis both in animals and humans [49-51]. The result of the acid load is therefore the decrease in urinary citrate.

Among the causative factors of hypocitraturia there is the low dietary consumption of fruit and vegetable. These foods increase the daily intake of alkalis that reduces the proportion of H^+^ allowing the binding to the trivalent citrate and therefore reducing citrate tubular reabsorption [52]. A diet rich in fruits and vegetables is now part of the nutritional advices that are provided to patients not only for the effect on citraturia, but also because it helps to increase the volume of urine, provides alkaline potassium and magnesium, increases the urinary pH [53] and finally implies a lower content of animal protein and salt [46]. In 2010, Taylor and co-workers found that a DASH – style diet was associated with an increased urinary output, regardless of fluid intake [54]. They speculated that higher urinary volumes were a result of the higher food water content and they reported that a high dietary intake of fruits and vegetables was linked to increased urinary citrate levels and higher urine pH. Several studies attribute the anti-lithogenic effect of vegetables to the mechanisms above listed.

## Conclusions

An isolated episode of lithiasis in adulthood may be considered occasional. Many patients, however, keep on forming stones during their lives, with greater or lesser frequency. This can be associated only with the presence of stable predisposing factor, such as to cause a net increase in the risk of recurrence. In these situations, once solved any acute problem related to the presence of calculi in the urinary tract, the specialist must investigate the metabolic situation, in order to be able to provide detailed advice. However, we recall that the far more important role in preventing is that performed by dilution of urine with high introduction of liquids. We would also emphasize the importance of nutritional management of stone formers patients, through the introduction of a diet aimed to prevent or at least to delay recurrences. In fact, in these patients, who have a genetic background of increased lithogenic risk, it is possible that dietary mistakes can have more influence than in non-predisposed subjects.

## Abbreviations

NADC: Sodium dependent carrier; DASH diet: Dietary approaches to stop hypertension

## Competing interests

The authors declare that they have no competing interests.

## Author's contributions

ED, GV, AN, and LS have drafted the manuscript. TM, AT, AM, CB, BR, and DC have all contributed substantially to the manuscript. All authors have read and approved the final manuscript.
